# The Conserved Family of the Pyridoxal Phosphate-Binding Protein (PLPBP) and Its Cyanobacterial Paradigm PipY

**DOI:** 10.3390/life12101622

**Published:** 2022-10-17

**Authors:** Lorena Tremiño, Antonio Llop, Vicente Rubio, Asunción Contreras

**Affiliations:** 1 Dto. de Fisiología, Genética y Microbiología, Universidad de Alicante, 03690 San Vicente del Raspeig, Spain; 2 Instituto de Biomedicina de Valencia, Consejo Superior de Investigaciones Científicas (IBV-CSIC), 46010 Valencia, Spain; 3 Group CB06/07/0077 at the Instituto de Biomedicina de Valencia (IBV-CSIC) of CIBERER-ISCIII, Centro de Investigación Biomédica en Red de Enfermedades Raras, 28029 Madrid, Spain

**Keywords:** cyanobacteria, nitrogen regulation, COG0325, PLPHP, PLPBP, PipY, YggS, pyridoxal phosphate, *Synechococcus elongatus* PCC7942, vitamin B_6_-dependent epilepsy

## Abstract

The PLPBP family of pyridoxal phosphate-binding proteins has a high degree of sequence conservation and is represented in all three domains of life. PLPBP members, of which a few representatives have been studied in different contexts, are single-domain proteins with no known enzymatic activity that exhibit the fold type III of PLP-holoenzymes, consisting in an α/β barrel (TIM-barrel), where the PLP cofactor is solvent-exposed. Despite the constant presence of cofactor PLP (a key catalytic element in PLP enzymes), PLPBP family members appear to have purely regulatory functions affecting the homeostasis of vitamin B_6_ vitamers and amino/keto acids. Perturbation of these metabolites and pleiotropic phenotypes have been reported in bacteria and zebrafish after *PLPBP* gene inactivation as well as in patients with vitamin B_6_-dependent epilepsy that results from loss-of-function mutations at the *PLPBP*. Here, we review information gathered from diverse studies and biological systems, emphasizing the structural and functional conservation of the PLPBP members and discussing the informative nature of model systems and experimental approaches. In this context, the relatively high level of structural and functional characterization of PipY from *Synechococcus elongatus* PCC 7942 provides a unique opportunity to investigate the PLPBP roles in the context of a signaling pathway conserved in cyanobacteria.

## 1. Introduction

Cyanobacteria, phototrophic organisms performing oxygenic photosynthesis, constitute an ecologically and biotechnologically important phylum, responsible for the evolution of the oxygenic atmosphere, being the main contributors to marine primary production [1]. Their photosynthetic lifestyle and ease of cultivation make them ideal production systems for several high-value compounds, including biofuels [2]. Despite important breakthroughs in the genetic analysis of cyanobacteria, there is still a remarkable proportion of genes of unknown function in this phylum, many of which are presumably relevant to the biology of cyanobacteria.

The cyanobacterium *Synechococcus elongatus* PCC7942 (hereafter *S. elongatus*), the only photosynthetic organism for which the contribution of each gene to fitness has been evaluated so far [3], is being used as a model system to address fundamental questions concerning the photosynthetic lifestyle. More recently, the *S. elongatus* genome has been used as the reference organism to create a database for the Cyanobacterial-Linked Genome [4], accessible through an interactive platform “https://dfgm.ua.es/es/cyanobacterial-genetics/dclg/index.htm (accesed on 1 August 2022)”. 

In bacteria and plants, 2-oxoglutarate (2-OG), a key metabolic signal of the intracellular carbon-to-nitrogen balance, is sensed by the highly conserved and widely distributed signal transduction protein PII. PII regulates the activity of proteins involved in nitrogen metabolism by direct protein–protein interactions [5]. In *S. elongatus* PII interacts with a small (89 residues) protein called PipX (PII-interacting protein X), which was initially identified in yeast two-hybrid analyses [6,7].

PipX was also found in searches for proteins interacting with NtcA, the global transcriptional regulator involved in nitrogen assimilation in cyanobacteria [8]. PipX stabilizes the conformation of NtcA which is transcriptionally active and probably helps the local recruitment of RNA polymerase to NtcA-dependent promoters [9]. At low 2-OG concentrations corresponding to nitrogen-excess conditions, the sequestration of PipX by PII renders PipX unavailable for NtcA binding and activation, reducing the expression of NtcA-dependent gene targets [9,10,11,12,13]. Partner swapping by PipX is enabled by its N-terminal Tudor-like domain (TLD/KOW), which provides contacts for both NtcA and PII. Complex formation with PipX increases the affinity of PII for ADP [9], and, conversely, the interaction between PII and PipX is highly sensitive to fluctuations in the ATP/ADP ratio [14]. Thus, PipX partner swapping between PII and NtcA integrates signaling of the carbon-to-nitrogen ratio and the energy status by PII with the regulation of nitrogen-responsive genes controlled by NtcA [10,15,16].

Interestingly, a high PipX/PII ratio prevents growth [11,17] and, consistent with this, cyanobacterial genomes always contain at least as many copies of *glnB* as of *pipX* [18], suggesting that a relatively high ratio of PII over PipX is required to counteract unwanted interactions with low-affinity PipX partners.

In *S. elongatus pipX* is co-transcribed with the downstream gene *pipY*. This last gene belongs to the widely distributed and highly conserved pyridoxal phosphate (PLP)-binding protein (COG0325/PLPBP) family that is involved in vitamin B_6_ and amino acid homeostasis [19]. The PLPBP family (also termed ProsC/PROSC or COG0325 family) members are found in all kingdoms of life, exemplified by the proteins YBL036C (yeast), YggS (Gram-negative bacteria), YlmE (Gram-positive bacteria), PipY (cyanobacteria), PLPHP (humans) and HTH5 (rice). These are all single-domain proteins exhibiting the fold type III of PLP-holoenzymes [20,21,22,23,24] with no known enzymatic activity. 

The association of *pipY* with *pipX* in an operon provides a unique opportunity to investigate the roles of PLPBP proteins in the context of a signaling pathway conserved in cyanobacteria, which is interconnected with the well-characterized nitrogen regulation network [10]. Importantly, PipY from *S. elongatus* is one of the best-characterized PLPBP members and can be regarded as a paradigm for PLPBP proteins [19,22,25].

## 2. Structural and Functional Features of PLPBPs

### 2.1. PLP Is Solvent-Exposed in PLPBP Structures 

The vitamin B_6_ vitamer PLP is used as a cofactor for enzyme-catalyzed reactions which include transamination, decarboxylation, racemization, aldol cleavage, or replacement reactions among others [26]. Since amino acid metabolism and other essential processes require PLP-dependent enzymes [27,28], PLP availability is of paramount importance to supply cofactors to activate newly synthesized apo-B_6_ enzymes. PLP is also required as a cofactor of glycogen phosphorylase [29] and certain transcriptional factors and regulators [28]. However, its aldehyde group endows PLP with high chemical reactivity, sometimes causing the inactivation of proteins (see for example, [30]), and therefore additional mechanisms are required for keeping the levels of free PLP low in cells and tissues. In the first report of a member of this family, Eswaramoorthy et al. (2003) documented structural parallelisms between the yeast protein YBL036C and the N-terminal domain of alanine racemases, leading them to infer (and even to provide some experimental hints for it) that PLPBP had alanine racemase activity [21]. However, no amino acid racemase, decarboxylase, deaminase, or transaminase activities were found for *E. coli* or human proteins [31], and although crystal structures of alanine racemase with bound substrates (D-ala) or inhibitors (D-cycloserine) have been determined [32], extensive crystallization attempts with these molecules did not detect any binding to PipY [22]. Furthermore, in vivo work did not support alanine racemase activity for *S. elongatus* PipY [19]. Therefore, despite the key importance of the PLP cofactor for PLPBP function (see below), PLP appears to have no catalytic function in the PLPBP family. 

**Figure 1:**
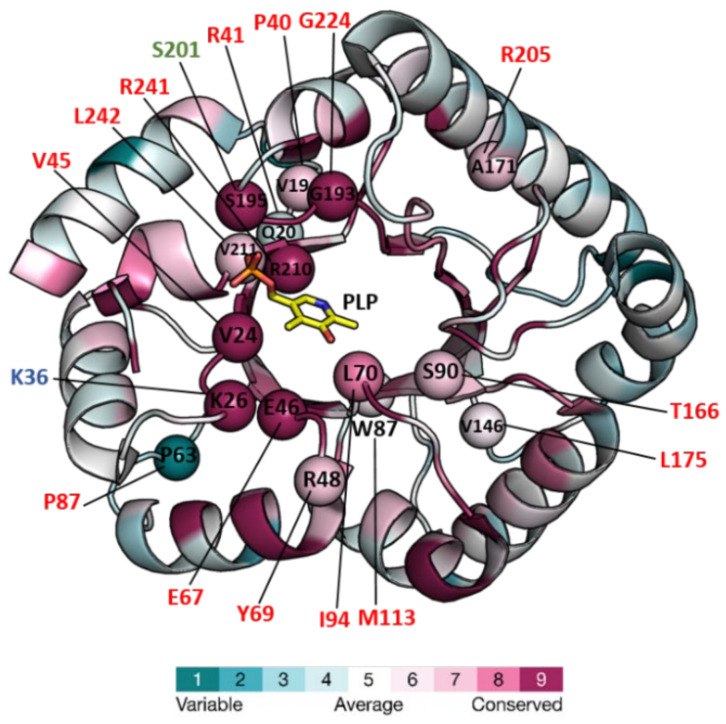
Structure of PipY from *S. elongatus* (PDB 5NM8) colored for the evolutionary conservation of residues among PLPBP homologs, and mapping therein, residues targeted by missense mutations. The structure is in a cartoon representation except for the PLP, which is in a stick representation with C, O, N, and P atoms in yellow, red, blue, and orange, respectively. Color-coding of the structure from cyan to magenta according to the residue conservation score (the higher, the more conserved) given by The ConSurf Server “URL https://consurf.tau.ac.il/consurf_index.php (accesed on 3 August 2022) when queried with chain A of the PDB 5NM8, with default parameters. Spheres mark the location in PipY of known human PLPHP mutations (see Table 2). Residue numbers are given in one letter code, in black for *S. elongatus*, and shown in red, green, and blue, the human mutations causing vitamin B_6_-dependent epilepsy, and the in vitro mutations obtained in the corresponding proteins of *F. nucleatum,* and *E. coli*, respectively.

Structures of six PLPBP members have been determined and deposited in the Protein DataBank (PDB, “https://www.rcsb.org/ (accesed on 1 September 2022)”) (Table 1). All of these structures correspond to single-domain chains folded according to the triose phosphate isomerase (TIM) barrel typically found in the fold type III of PLP-dependent enzymes. The only ones reported to date from a eukaryotic organism correspond to yeast protein YBL036C. The others are from a Gram-positive bacterium (*Bifidobacterium adolescentis*), and four Gram-negative bacteria including the cyanobacterium *S. elongatus* (Table 1). *S. elongatus* PipY structures with and without PLP offer high resolution and have been used to estimate the effects of clinical missense mutations found in the *PLPBP* human gene in patients with vitamin B_6_-dependent epilepsy [22,23]. Here, we use PipY as a reference for the additional discussion on structural and functional details concerning studied members of the protein family. Figure 1 shows the structure of PipY containing PLP (PDB file 5NM8). 

The TIM-barrel fold, initially described for triosephosphate isomerase [33], is a highly widespread protein fold, generally reported as consisting of eight α helices that alternate with parallel β strands of a circularly closed β-sheet, in which the helices encircle the sheet (reviewed in [34]). The TIM-barrel of PLP proteins, first described for alanine racemase [32], characterizes the fold type III of PLP-dependent enzymes and presents an extra N-terminal α helix preceding the first of the eight βα repeats. However, while this modified TIM-barrel is part of a two-domain subunit forming homodimers in alanine racemase, ornithine decarboxylase, and the broad specificity amino acid racemase [20,26,35], PLPBP members are single-domain proteins that appear to be mainly monomers (but see discussion below) ([21,22]; and other PDBs in Table 1). 

**Figure 2:**
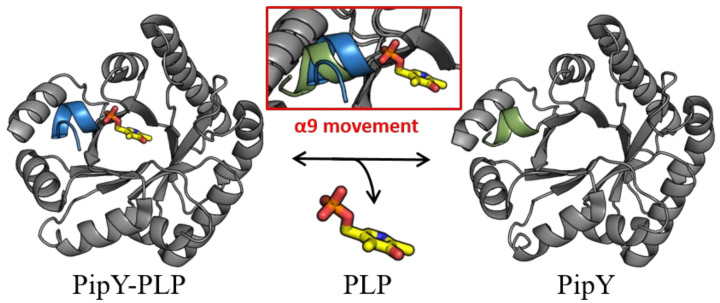
PipY structures with and without PLP illustrate the two positions of helix 9. Cartoon representation of PipY structure from *S. elongatus* complexed with PLP (PDB 5NM8) and PipY-Apo form (PBD 5NLC), with α-helix 9 shown in blue and green, respectively. Inset: Displacement of α-helix 9 observed in the PLP-containing form is highlighted by superimposing both protein forms. The PLP molecule is illustrated using stick representation, where C, O, N, and P atoms are colored yellow, red, blue, and orange, respectively.

While PLP is found in PLPBP structures in the same location that it is found in the fold type III of PLP enzymes, in all PLPBP structures the PLP cofactor is solvent-exposed and highly accessible, thus being appropriately positioned for a role of PLPBP as a PLP delivering device in cells. In this context, the role of the C-terminal α helix (helix 9) of PipY in anchoring the phosphate of PLP, and the relatively large changes in helix 9 orientation depending on the presence or absence of PLP have led to the hypothesizing [22] that this helix may have a role in being a trigger for the binding and release of PLP (Figure 2). 

The available data so far suggest that the proteins of this family might act as PLP carriers which supply the cofactor to PLP-dependent enzymes, shielding this cofactor from unwanted reactions with other molecules, although this has not been strictly proven and the mechanisms involved remain unclarified.

### 2.2. Dimerization of Just Some PLPBP Family Members?

While all available crystal structures are consistent with PLPBP family members being monomers, size-exclusion chromatography of human PLPHP, performed in two different studies [23,36] revealed a second peak corresponding to dimers. In [23], the minor peak was shown to depend on disulfide bridges, a result interpreted as PLPHP being mainly monomeric with the possibility of stable dimerization via the formation of a disulfide bridge between exposed cysteines. Consistent with this, human mutation Tyr69Cys increased dimer formation (Table 2). However, Fux and Sieber [36] challenged this view, reporting that their PLPHP preparation was predominantly dimeric even under reducing conditions [36]. They also suggested that discrepancies with the previous work may be due to differences in expression strains or purification strategies. It is worth noting that while the human protein contains five cysteine residues, the amino acid chains of *S. elongatus* PipY and *E. coli* YggS have just one or two cysteines, respectively, and thus they would be less prone to making disulfide bonds under oxidative conditions.

Although the relative importance of dimeric forms of PLPBP family members in cell systems remains to be determined, it is worth noting that dimer formation could significantly enhance the putative function of PLPBPs in shielding PLP transport or storage, which are the most likely functions attributed to PLPBPs so far. Importantly, the identification of dimeric forms of human PLPHP opens the possibility that at least two different forms of PLPBPs (monomeric and dimeric), perhaps with different regulatory properties, might be found in cells. Whether dimer formation is a property of just some PLPBP members or a universal feature of the family, and whether in vivo dimer formation requires cysteines in critical positions or can also be induced by other effectors or cell components are open questions that require further investigation. 

## 3. PLPBP-Related Phenotypes

### 3.1. Null Mutations, Heterologous Complementation, and Animal Disease Models Support Universal Functions of PLPBP Family Members

**Figure 3:**
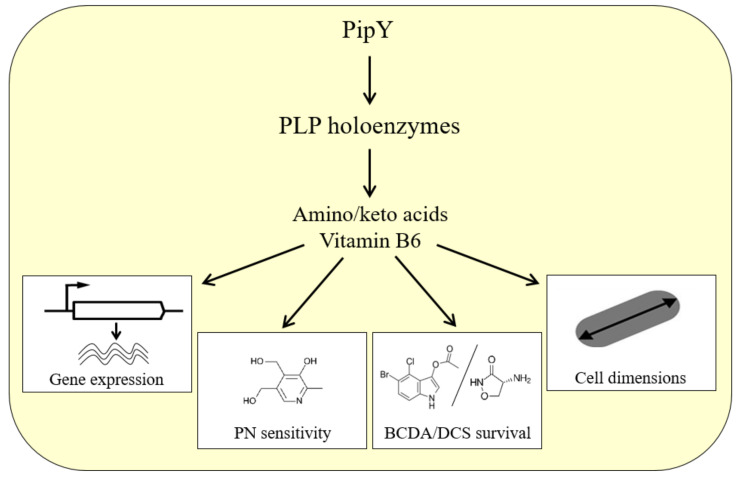
Roles of PipY and affected processes in cyanobacteria. PipY would function as a PLP storage and delivery module, and influence the activity of PLP-holoenzymes, which in turn affect the amino/keto acid pool and the processes responsive to (some of) these metabolites. Phenotypes dependent on PipY include the control of gene expression, PN sensitivity, survival in the presence of antibiotics targeting PLP-holoenzymes, and altered cell dimensions. Abbreviations: PN, pyridoxine; DCS, D-cycloserine.

Consistent with a key role of PLPBPs in PLP homeostasis, inactivation of the corresponding genes results in alterations of the relative levels of B_6_ vitamers and amino/keto acids in all organisms investigated. The null mutants of *E. coli*, *S. elongatus, Salmonella enterica, Acidovorax citrulli*, *S. cerevisiae*, and *Danio rerio* (zebrafish) showed sensitivity to pyridoxine (PN), an overaccumulation of PNP and/or an imbalance of the amino/keto acid pools ([37] and references within it). As a consequence of these metabolic alterations, in which PLP-dependent enzymes are presumably involved, pleiotropic phenotypes have been associated with PLPBP deficiency, which may alter different cellular processes in different organisms. The phenotypic changes observed (at least) in cyanobacteria are summarized in the general model for PLPBP function illustrated in Figure 3.

As an example, *S. elongatus* *pipY* and *A. citrulli yggS* null mutants both show high sensitivity to the antibiotic β-chloro-D-alanine (BCDA), while *S. elongatus pipY* mutants are also sensitive to D-cycloserine (DCS) [19,38]. Importantly, PipY overexpression did not increase resistance to either of these antibiotics, as would be expected for a direct antibiotic target. Instead, PLPBPs indirectly confer antibiotic resistance by protecting the essential, high-affinity antibiotic targets specific for their cognate PLP-dependent enzymes. The specific targets in this example are glutamate racemases for BCDA [39] and alanine racemases for both DCS and BCDA [40]. The indirect role of antibiotic resistance also agrees with the reported failure to obtain crystals of PipY with D-ala or DCS [22].

Multiple lines of evidence reinforce the idea that diverse PLPBPs share common functions in cells. Distantly related PLPBP members were able to rescue strain-specific defects, such as Val overproduction and the PN sensitivity phenotypes of *E. coli yggS* mutants. These two *E. coli yggS* mutant phenotypes were both rescued by the heterologous expression of orthologs from bacilli, yeast, plants (*Zea mays*, *Arabidopsis thaliana*), or humans [31,41,42]. The introduction of human PLPHP into the corresponding null mutants of yeast partially rescued the growth phenotype on poor carbon sources [43]. Last but not least, PLPHP deficiency reproduced the human disease in zebrafish larvae; the knockout mutants showed multiple signs of seizure activity before dying, while pyridoxine treatment improved the epileptic phenotype and prolonged lifespan [43].

### 3.2. PLPBP Mutations Cause B_6_-Dependent Epilepsy in Humans

Relatively recently, recessive mutations affecting human *PLPBP* were reported as causal in patients with vitamin B_6_-dependent epilepsy [41]. Since then, many others have reported familial cases of vitamin B_6_-dependent epilepsy associated with missense, nonsense, and splice-site mutations at the *PLPBP* locus [43,44,45,46,47,48,49,50,51,52,53,54]. Some of the PLPHP mutant proteins have been studied *in vitro* and found to decrease protein levels or PLP cofactor-binding [22,23,41], and Table 2. Furthermore, the missense mutations found in patients target highly conserved residues (except for residues Pro87 and His275), as shown in Figure 1, and Table 2.

As already indicated, most mutations decreased the binding of the PLP cofactor, protein levels, and/or thermal stability. That is the case for mutations Pro40Leu, Tyr69Cys, Pro87Leu, Arg205Gln, Leu175Pro and Arg241Gln [22,23], Arg41Gln, Val45Asp and Glu67Lys [36] and Ile94Phe, Thr116Ile and Gly224Ala [43]. It is worth noting that although Pro87 is a variable residue, the restriction in chain stereochemistry imposed by the imino acid nature of proline explains the frequently drastic structural effects resulting from proline substitutions. Thus, it is not surprising that despite the lack of conservation, Pro87Leu also results in the loss-of-function of PLPHP. On the other hand, His275Asp targets the non-conserved C-terminal extension of the human protein and it is unlikely to directly affect PLP binding, raising questions on the function of this extension. 

In summary, the reported mutations at the *PLPBP* locus causing vitamin B_6_-dependent epilepsy are all consistent with the loss-of-function of PLPHP. To our knowledge, no gain-of-function mutations or mutations consistent with increased expression of PLPHP have been reported, raising the question of whether such mutations would result in a pathological consequence or cause a different type of disease pathology.

**Table 2:** Missense mutations reported in human PLPHP associated with vitamin B6-dependent epilepsy, as well as two experimental mutations in orthologous bacterial proteins. Molecular mechanisms of damage.

Amino Acid Change in HuPLPHP						Molecular Mechanism: Effect on PLPBP Protein		
Amino acid	Conserv. Score ^1^	Change	Clinical effects	Reported in	Number of patients	Observed effect	Inferred from	Ref.
P40	7	P40L	Seizures	[44] [51]	1 (P40L/R241Q) 1 (P40L/splicing)	↓ thermostability	In vitro studies on rHuPLPHP	[23]
R41	4	R41Q	Seizures Mild disease ^2^	[54] [43]	2 (homozygous;R41Q/V45D) 3 (homozygous)	↓yield/misfolding? ↓thermostability	In vitro studies on rHuPLPHP	[36]
		R41W	Seizures, death	[50]	1 (homozygous)	NT	NT	NT
V45	9	V45D	Seizures	[54]	1 (R41Q/V45D)	↓↓PLP content ↓ thermostability	In vitro studies on rHuPLPHP	[36]
K47	9	K47A	Not reported in humans (prenatally lethal?)			lack of PLP	rEcyggS^K36A^	[31]
E67	9	E67K	Seizures Severe disease ^2^	[54] [43]	1 (homozygous) 3 (homozygous)	Misfolding	In vitro studies on rHuPLPHP	[36]
Y69	7	Y69C	Seizures Moderate disease ^2^	[44]	1 (homozygous)	Higher dimerization ↓PLP content	In vitro studies on rHuPLPHP	[23]
P87	1	P87L	Seizures Severe disease ^2^	[41] [44] [53]	1(P87L/R241Q) 1 (homozygous) 1 (P87L/splicing)	↓solubility/misfolding	In vitro studies on rHuPLPHP	[23]
I94	8	I94F	Seizures Mild disease ^2^	[43]	1 (homozygous)	Proposed ↓ in PLP saturation	Structural modeling of HuPLPHP	[43]
M113	6	M113T	Seizures	[51]	1 (M113T/C15X)	NT	NT	NT
T116	7	T116I	Seizures Severe disease ^2^	[43]	2 (1 homozygous; 1 homozygous (T116I/H275D))	Proposed ↓ in PLP saturation	Structural modeling of HuPLPHP	[43]
L175	6	L175P	Seizures Severe disease ^2^	[41]	1 (homozygous)	Misfolding	In vitro studies on rHuPLPHP	[23]
R205	7	R205Q	Seizures Moderate disease ^2^	[44] [54]	1 (R205Q/null) 1 (homozygous)	↓thermostability	In vitro studies on rHuPLPHP	[23]
G224	9	G224A	Seizures Severe disease ^2^	[43]	1 (G224A/splicing)	Proposed ↓ in PLP saturation	Structural modeling of HuPLPHP	[43]
S226	9	S226A	Not reported in humans (prenatally lethal?)			↓PLP saturation	rFn^S201A^	[24]
R241	9	R241Q	Seizures	[41] [44] [52]	1 (P87L/R241Q) 1 (P40L/R241Q) 1(R241Q/splicing)	↓solubility ↓thermostability ↓PLP binding	In vitro studies on rHuPLPHP In vitro studies on rSePipY^R210Q^	[22,23]
I242	7	I242T	Seizures	[45]	1 (homozygous)	NT	NT	NT
H275	NA	H275D	Seizures	[43]	1 (homozygous T116I/H275D)	Variant of uncertain significance (VUS)	Structural modeling of HuPLPHP	[43]

NA: not applicable. NT: not tested. HuPLPHP: Human PLPHP. rHuPLPHP: recombinant human PLPHP. rEcyggS^K36A^: recombinant *E. coli* yggS K36A mutant. rSePipY^R210Q^: recombinant *S. elongatus* PipY R210Q mutant. rFn^S201A^: recombinant *F. nucleatum* yggS S201A mutant. ^1^ Conservation score as given in Figure 1. ^2^ Severity score given in [43].

### 3.3. Phenotypes Associated with PLPBP Excess

There are, to the best of our knowledge, three reports dealing with the phenotypic effects of increasing the levels of proteins in this family. In two of them, bacterial cells were engineered for PLPBP overexpression to address specific questions in the context of neighbor genes (see Section 4.1). However, the third report constitutes a unique example of an adaptative spontaneous mutation in a cereal crop, dealing with a natural variation in wild rice (*Oryza rufipogon*) conferring high-temperature tolerance at the phenological heading stage [55]. A map-based cloning approach for heat tolerance specifically at the panicle development stage identified *qHTH5* as a major quantitative trait locus (QTL) and the target gene (encoding a PLPBP ortholog) was named *HTH5*. The corresponding change was an up promoter mutation that increased transcript levels from *HTH5*. Although the exact mechanism of the heat tolerance phenotype was not ascertained, the authors suggested that it could be related to altered reactive oxygen species (ROS) homeostasis, thus expanding the putative functions of PLPBP.

It is worth noting that there are no studies so far dealing with the metabolic consequences of increased cellular levels of PLPBP family proteins, in contrast with the numerous reports describing metabolic changes corresponding to loss-of-function mutations in bacterial systems and human patients.

### 3.4. Synthetic Lethality between PLPBP Family Members and PLP-Holoenzymes Supports Some Functional Redundancy

Synthetic lethality between PLPBP and PLP-holoenzymes has been reported in *E. coli* for *yggS* with both *glyA* [42,56] and *serA* [57], in *S. enterica* for *yggS* and *aspC* [58] and *S. elongatus* for *pipY* and *cysK* [19]. Synthetic lethality probably reflects the common involvement of the corresponding protein pairs in amino acid and PLP homeostasis, but also supports the idea that any relatively abundant PLP-dependent protein (in addition to PLPBP family members) could fulfill a role as a PLP reservoir to prevent the possible consequences of an excess of unbound PLP. In line with this, PipY and CysK, the latter encoded by a non-essential gene with three paralogs in *S. elongatus*, are the two most abundant PLP-binding proteins in this cyanobacterium [19]. It is worth noting that PLPBPs appear to be relatively abundant proteins in many organisms, as is also the case with yeast or HeLa cells, where levels of PLPBPs are almost 10-fold higher compared to the median protein copy number [59].

Functional redundancy between PLPBP family members and PLP-holoenzymes, supported by synthetic lethality in distantly related bacterial groups, suggests that at least some of the multiple PLP-containing proteins expressed in living cells participate in PLP homeostasis. It also explains that, despite the universality of PLP-derived challenges in all types of cells, PLPBP members are neither essential nor ubiquitous in the cellular systems characterized so far [42]. Functional redundancy among PLP-holoenzymes appears to occur even in organisms with relatively small genomes, such as *S. elongatus*, where out of 41 PLP-binding protein sequences [60], 11 corresponded to non-essential and 6 to beneficial genes (their inactivation slows the growth of cultures) under standard photoautotrophic conditions [3]. 

## 4. Guilty by Association Strategies to Get Insights into PLPBP Family Functions

The lack of knowledge of the molecular mechanisms involved in the cellular functions of PLPBPs has probably contributed to the use of guilt by association strategies to establish connections between PLPBP members and specific biological processes. Gene synteny, and more recently protein interaction approaches have provided useful information in this context. 

### 4.1. Genes of the PLPBP Family in Clusters and Operons from Bacteria

Bacterial genomes offer an opportunity to apply the principle of guilt by association to search for characterized proteins that may belong to metabolic or signaling pathways involving PLPBP members. In this context, the distribution of *PLPBP* genes and physical clustering with genes of known function in bacteria was first investigated and discussed in detail by [42]. In addition to the association with *proC* (Pyrroline-5-carboxylate reductase, EC 1.5.1.2) [61], which is responsible for the *prosC* denomination [62,63], *PLPBP* genes also clustered with cell division and cell wall biosynthesis (*dcw*, in Gram-positive bacteria and mycobacteria), PLP salvage, surface motility, secretion, amino acid metabolism, and translation genes [42].

In *S. elongatus* and a majority of the available cyanobacterial genomes, the genes *sepF* (involved in cell division and restricted to Gram-positive bacteria and cyanobacteria) and *proC* were found at short distances downstream of *pipY*, while the arrangements *pipY-sepF* and *sepF-proC* were also frequently found in the available cyanobacterial genomes. The precise arrangement of *pipY-sepF-proC* and the linkage to the phylum-restricted gene *pipX* are both cyanobacterial hallmarks [19].

Several reports explored a putative role for PLPBP family members in cell division and cell wall functions by describing cell size or cell wall-related phenotypes in null or overexpressing strains. *Streptococcus pneumoniae ylmE* cells were slightly larger than normal [64], while normal cell dimensions were reported for *E. coli yggS* [42] and *S. elongatus pip*Y under standard culture conditions. On the other hand, overexpressing YlmE in *B. subtilis* blocked biofilm formation [65], while the overexpression of PipY increased *S. elongatus* cell length up to 28% [19]. Taken together, these findings support the notion that, at least in some bacterial groups, perturbations of the amino/keto acid pool may result in the accumulation of metabolic signals affecting cell wall metabolism or cell division. In addition, the possibility of physical interaction between a given PLPBP family protein and cell wall-related proteins cannot be excluded at present.

Very recently, it has been noted that the linkage with *pilT*, encoding an ATPase mediating pilus retraction and disassembly, is typical of phytopathogenic bacteria, where *yggS* and *pilT* are contiguous and divergently transcribed [38]. Importantly, *yggS* inactivation in *Acidovorax citrulli* significantly affected type III secretion, reduced swimming motility, and resulted in attenuated virulence. Thus, despite the lack of molecular details, the functional connection established between the *yggS* and *pilT* genes of phytopathogenic bacteria also supports the use of synteny approaches to gain additional insights into the role of PLPBP family members in particular biological processes or environmental contexts. 

### 4.2. The Close Relationship between PipX and PipY in Cyanobacteria

Since most of the mRNA transcripts identified in cyanobacteria are monocistronic (approximately 62% in *S. elongatus*), co-transcription of *pipX* and *pipY* is a strong indication of functional association [66,67]. Furthermore, close to 80% of *pipX* genes are found adjacent to *pipY* genes, presumably as part of bicistronic *pipX*Y operons. In addition, tight co-regulation and even translational coupling can be inferred by the relatively short or non-existent intergenic distances found between contiguous *pipX* and *pipY* coding sequences, strongly suggesting a functional interaction between PipX and PipY in most, if not all, cyanobacteria [19]. When not adjacent, as in *Synechocystis* strains, *pipX,* and *pipY* genes appear to be monocistronic or with no clear linkage to particular genes [25]. Another indication of the tight relationship between *pipX* and *pipY* genes in cyanobacteria is the finding that PipX increases the expression of either *pipY* or a reporter gene occupying the *pipY* locus in *S. elongatus,* suggesting the importance of the PipX-PipY balance in cyanobacteria [25].

While all this is strongly suggestive of physical interaction between PipX and PipY proteins in cyanobacteria, no evidence could be obtained for this interaction using yeast two-hybrid assays [19]. However, false negatives are not rare in this genetic system [6,68], and interactions between PipX and PipY proteins may depend on the factor(s) present in *S. elongatus* but absent from yeast nuclei, or present only under certain physiological or environmental conditions. As noted above, PipX is part of a complex partner-swapping network governed by molecule effectors signaling the ATP/ADP ratio (PII), 2-OG levels (PII, NtcA), or the GTP/GDP ratio (EngA) [10,16,69]. The levels of these metabolites and the relative abundance of the PipX interactants will influence the formation of complexes with different partners, raising the question of whether the putative PipX-PipY complexes would depend on an additional effector, and if the role played by PLP is as a cofactor of PipY, questions that deserve further investigation.

### 4.3. PLPHP Cellular Interaction Network

Co-immunoprecipitation with PLPHP after the crosslinking of proteins from a human embryonic kidney cell line (HEK293) revealed the presence of PLPHP in both the cytosol and mitochondria [36]. Contrary to the author’s expectations, an overrepresentation of PLP-dependent enzymes was not observed. Instead, a high number of proteins involved in cytoskeleton organization was detected, including components of the γ-tubulin ring complex necessary for microtubule nucleation at the centrosome, proteins involved in centriole and spindle formation, or several subunits of the f-actin capping complex which regulates the growth of actin filaments. The significance of these findings remains to be addressed.

## 5. Regulation of Gene Expression by PLPBP 

To our knowledge, there are only two reports analyzing differentially expressed transcripts in PLPBP protein-deficient mutants. The first one investigated the genetic interactions between PipX and PipY (see below). A more recent study presented results obtained from the *yggS* mutant of *A. citrulli* in the context of virulence and other related phenotypic features [38]. 

Another study investigated the effect of *PLPBP* inactivation on the proteome of the HEK293 cell line, which resulted in the significant upregulation of proteins associated with cytoskeleton organization and cell division as well as downregulation of two PLP-dependent enzymes involved in H_2_S synthesis [36].

While these studies are not easily comparable, the relatively high number of differentially expressed transcripts in bacteria and the proteins in the human cell line study emphasize the importance of PLP homeostasis in the global regulation of gene expression in the different cellular systems analyzed. 

Role of PipY in the Context of Cyanobacterial Nitrogen Regulation

The apparent recruitment of PipY, via PipX, into the 2-OG-dependent nitrogen interaction network of *S. elongatus* [10] provides a unique opportunity to investigate the functions of PipY, a paradigm PLPBP member, in the context of a relatively well-characterized signaling network in a cyanobacterial model system. 

Transcriptomic analysis with *S. elongatus pipX* null and gain-of-function mutant derivatives showed that NtcA targets were only a minor fraction of the affected transcripts [70] and are consistent with PipX having a role as a repressor of many photosynthesis and translation-related genes. The model emerging from this analysis is that PipX participates in at least four types of regulatory complexes, including among them the already characterized transcriptional complex NtcA-PipX. Importantly, transcriptomic analysis with single and double *pipX* and *pipY* null mutants revealed a PipY-dependent induction of NtcA-activated transcripts as well as the implication of both PipX and PipY in the regulation of NtcA-independent transcripts [19], where they could have similar or opposite effects, suggesting a rather complex regulation. The only other transcriptional regulator known to interact with PipX is PlmA [71], which so far is the best candidate to mediate one of the PipX-regulated transcriptional responses independent of NtcA. However, signals regulating PlmA activity remain to be discovered.

PipY may alter nitrogen signaling in cyanobacteria by affecting the levels of amino/keto acids, including 2-OG, a possibility suggested by the positive role of PipY at NtcA-activated transcripts, which are dependent on 2-OG. On the other hand, the expression of the NtcA-independent transcripts that are co-regulated by PipX-PipY would require a transcriptional regulator that can also respond to amino/keto acid effectors, or perhaps to PLP or related compounds, a possibility worth exploring.

## 6. Concluding Remarks

The structural and functional information gathered from different systems and heterologous complementation analyses of null mutants emphasize the importance of the conserved role of the PLPBP family in vitamin B_6_ vitamers and amino/keto acid homeostasis, providing a rationale for the pleiotropic phenotypes and changes in gene expression patterns caused by PLPBP deficiency. 

Despite growing interest in the field, recently fueled by the identification of familial mutations of the *PLPBP* causing vitamin B_6_-dependent epilepsy in humans, fundamental questions concerning the mechanism of action of PLPBPs remain to be answered and additional lines of investigation are required to advance knowledge. Bacterial systems can provide further insights into the function and conserved structural features of PLPBPs in cases where site-directed mutagenesis and genetic manipulation are rapid and tractable. The metabolic effects of *PLPBP* overexpression and relevant phenotypic effects of putative gain-of-function mutations should also be addressed. PLPBP members with tight functional connections with known genes also deserve attention. In this context, the tight association of *pipY* with *pipX*, encoding a key component of the cyanobacterial nitrogen interaction network, provides a unique opportunity to investigate the roles of the PLPBP family proteins in the context of a signaling pathway conserved in cyanobacteria that would provide valuable lessons on both universal and specific functions of this yet mysterious protein family. 

## Figures and Tables

**Table 1:** Structures of COG0325/PLPB family proteins were determined and deposited in the Protein DataBank (PDB).

Organism	Protein	PDB File	Vitamer	Ligands	Amino Acid Changes	Resolut. (Å)	Deposition Year	Ref.
*Escherichia coli*	YggS	1W8G	PLP	Isocitrate	None	2.00	2004	–
		3SY1	PLP	MES Acetate	L32V/G56S/N58H/H81N/ I83A/H102I/M165S/S202A/ M205Q/R221A hexamutant	1.47	2011	–
		7UBQ	PNP *	None	None	2.60	2022	–
		7UB4	PLP	None	K36A/K38A/K233A/K234	2.40	2022	–
		7UAX	None	PO_4_H_3_	K36A/K38A	2.07	2022	–
		7U9H	None	SO_4_H_2_	None	2.00	2022	–
		7UBP	PLP	SO_4_H_2_	K36A/K137A	2.30	2022	–
		7UB8	PLP	Butanediol	K38A/K137A/K233A/K234A	2.30	2022	–
		7UAU	PLP	SO_4_H_2_	K137A	2.10	2022	–
		7UAT	PLP	PO_4_H_3_	K36A	2.00	2022	–
		7U9C	PLP	PO_4_H_3_	None	2.10	2022	–
*Bifidobacterium* *adolescentis*	YggS	3CPG	PLP	Acetate	Se-Met **	1.71	2008	–
*Agrobacterium tumefaciens*	YggS	3R79	PLP	Acetate Pr^+3^	Se-Met **	1.90	2011	–
*Synechococcus elongatus*	PipY	5NLC	None	PO_4_H_3_	None	1.90	2017	[22]
		5NM8	PLP	Ca^2+^	None	1.93	2017	[22]
*Fusobacterium nucleatum*	YggS	7F8E	None	SO_4_H_2_	Se-Met **	2.08	2021	–
		6KZW	None	PO_4_H_3_	T5A/N202S, Se-Met **	2.08	2019	–
		7YGF	Structure not released			2.08	2022	[24]
*Saccharomyces cerevisiae*	YBL036C	1CT5	PLP	None	Se-Met **	2.00	1999	[21]
		1B54	PLP	None	None	2.10	1999	[21]

* Pyridoxine-5′-phosphate. ** Methionine replaced by selenomethionine—data not available.

## Data Availability

Not applicable.
